# Bivalirudin versus Heparin plus Glycoprotein IIb/IIIa Inhibitors in Women Undergoing Percutaneous Coronary Intervention: A Meta-Analysis of Randomized Controlled Trials

**DOI:** 10.1371/journal.pone.0169951

**Published:** 2017-01-17

**Authors:** Haiyan Xu, Bingjian Wang, Jing Yang, Shuren Ma, Xiongwei Xie

**Affiliations:** Department of Cardiology, Huai’an First People’s Hospital, Nanjing Medical University, Huai’an, Jiangsu, P. R. China; University of Bologna, ITALY

## Abstract

Bivalirudin has been shown to be safe and efficacious compared with heparin plus glycoprotein IIb/IIIa inhibitor (GPI) in patients undergoing percutaneous coronary intervention (PCI). Whether bivalirudin would have the beneficial effects in female patients undergoing PCI remains unknown. We searched the literature for randomized controlled trials that assessed bivalirudin versus heparin plus GPI therapy in female patients undergoing PCI. The primary efficacy end point was major adverse cardiovascular events (MACE) within 30 days. The secondary efficacy end points were 30-day incidence of all-cause mortality, myocardial infarction (MI), urgent/ischemia-driven revascularization of target vessel. The safety end point was major bleeding up to 30 days. A total of 4,501 female patients were included in five randomized trials. No significant difference in MACE emerged between bivalirudin and heparin plus GPI at 30 days (8.15% vs 8.76%, RR 0.94, 95% CI 0.77–1.16, *P* = .57). There were no significant differences in rates of mortality (1.28% vs 1.91%, RR 0.74, 95% CI 0.45–1.20, *P* = .22), MI (5.46% vs 5.25%, RR 1.02, 95% CI 0.79–1.32, p = .88), or target vessel revascularization (2.13% vs 1.65%, RR 1.43, 95% CI 0.88–2.30, *P* = .15). Compared with heparin plus GPI, bivalirudin was associated with a significant reduction in 30-day major bleeding (5.32% vs 9.20%, RR 0.58, 95% CI 0.47–0.72, *P* < .0001). In conclusion, bivalirudin is associated with a significant reduction in 30-day major bleeding without increased ischemic events compared with heparin plus GPI in female patients undergoing PCI.

## Introduction

Primary percutaneous coronary intervention (PCI) is a preferred reperfusion strategy for the treatment of ST-segment elevation myocardial infarction (STEMI) and is recommended by international guidelines [1]. However, adjunct antithrombotic therapy plays a crucial role in preventing adverse thrombotic events during and after primary PCI [2]. There is a wealth of literature have demonstrated that female gender possess a worse outcome in PCI related to periprocedural complications and bleeding compared to men [3–6]. Therefore, optimal antithrombotic therapy with decreased bleeding risks and without increased ischemic events is urgently needed for female patients undergoing primary PCI.

Bivalirudin, a direct thrombin inhibitor, is used instead of heparin plus a glycoprotein IIb/IIIa inhibitor (GPI) during PCI that has been proved to be associated with decreased rates of bleeding events [7–9]. However, the clinical benefits and safety of bivalirudin are still controversial. Whether women, who are at high risk for bleeding, receive bivalirudin during PCI resulted in a decrease in major bleeding and major adverse cardiovascular events (MACE) when compared to heparin plus GPI therapy in contemporary practice has not been determined.

To address the gap in knowledge, we performed a meta-analysis of all published randomized trials to evaluate the safety and efficacy of bivalirudin compared with heparin plus GPI in female patients undergoing PCI.

## Materials and Methods

### Search Strategy and Study Selection

The meta-analysis was performed according to the protocols recommended by the Preferred Reporting Items for Systematic Reviews and Meta-Analyses group for randomized controlled trials (PRISMA)[10]. Two investigators (HYX and XWX) did a computerized literature search of PubMed, Cochrane Library, EMBASE, and Clinical Trials. gov databases from inception until July 25, 2016 using the keywords “bivalirudin”, “Hirulog”, “Angiomax”, “heparin”, “Percutaneous Coronary Intervention”,“gender”, “sex”and “female”. Inclusion criteria were as following: (1) randomized controlled trials of bivalirudin versus heparin plus GPI; (2) patients undergoing PCI whether elective or urgent; (3) clinical outcomes were reported and follow-up time was more than 30 days; (4) reported subgroup analysis of outcomes in female.

The primary efficacy end point was MACE within 30 days. The secondary efficacy end points were 30-day incidence of all-cause mortality, myocardial infarction (MI), urgent/ischemia-driven revascularization of target vessel. MACE was defined as the composite of all-cause mortality, MI, along with urgent/ischemia-driven revascularization of target vessel. The primary safety end point was 30-day incidence of major bleeding.

### Data Extraction and Quality Assessment

Data were independently extracted by two investigators (BJW and SRM) and divergences were resolved by consensus. We performed objective assessment of the trials using a standardized form data abstraction instrument. We evaluated the studies' quality according to the Cochrane Collaboration guidelines (allocation concealment; random sequence generation; blinding of participants, personnel, and outcome assessors; incomplete outcome data; selective outcome reporting; other potential sources of bias)[11]. An investigator (JY) verified the data and analyzed the study quality.

### Statistical Analysis

All data were analyzed according to the intention-to-treat principle. Risk ratios (RRs) and 95% confidence intervals (CIs) were used as summary statistics. Random-effect model was used to assess the overall estimate. Heterogeneity was assessed by I^2^ test and its P value. I^2^ <25% was defined as low heterogeneity and >50% was defined as significant heterogeneity. Pre-specified subgroup analyses performed by type of patient enrolled (predominantly AMI or predominantly elective or urgent PCI), use of GPIs (provisional in the bivalirudin arm or not) and access site (femoral access or radial access). Serially left one study out was carried out to eliminate sources of heterogeneity in sensitivity analyses. A 2-tailed P < 0.05 was considered statistically significant. The potential publication bias was evaluated by constructing a funnel plot, Begg and Egger test. All analyses were performed using STATA software version 12 (STATA Corporation; College Station, Texas).

## Results

### Search Results, Baseline Characteristics

**Fig 1:**
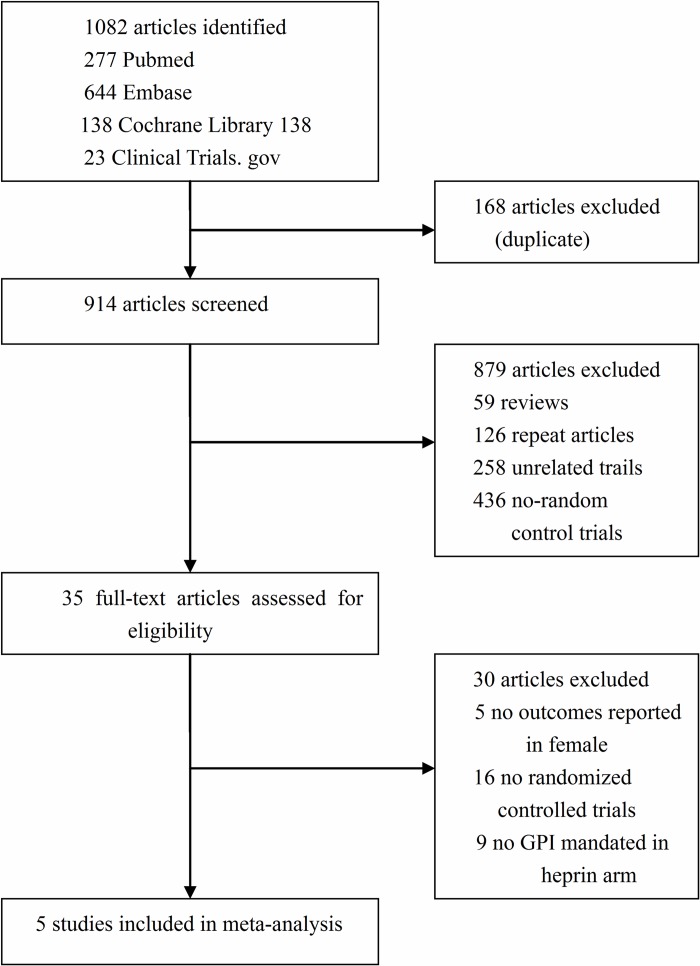
Study selection flow diagram. Summary of progress through the stages of search and eligible studies identify.

We identified 1082 potentially relevant articles from the initial literature search and five trials met all inclusion criteria [12–16]. The selected procedure is summarized in Fig 1. All these articles were subgroup analyses of the following trials: Bivalirudin in Acute Myocardial Infarction vs Heparin and GPI Plus Heparin Trial (BRIGHT), Harmonizing Outcomes with Revascularization and Stents in Acute Myocardial Infarction (HORIZONS-AMI), Intracoronary Stenting and Antithrombotic Regimen: Rapid Early Action for Coronary Treatment (ISAR-REACT-4), Acute Catheterization and Urgent Intervention Triage Strategy (ACUITY) and Randomized Evaluation in PCI Linking Angiomax to Reduced Clinical Events (REPLACE-2).

The characteristics of the included trials are detailed in Table 1. Of the 4,501 randomized female patients, 2,196 were assigned to bivalirudin treatment and 2,305 were assigned to heparin plus GPI treatment. Except for REPLACE-2, all the four trials enrolled female patients with AMI undergoing PCI. A population of lower-risk ACS patients undergoing urgent or elective PCI were included in REPLACE-2 trial. In BRIGHT, 131 patients received a GPI (tirofiban) in heparin plus GPI arm, while 134 patients only received heparin. Abciximab or enoxaparin was plannned used in all patients of heparin plus GPI arm in the other four trials. A provisional GPI was used in patients of bivalirudin arm in HORIZONS-AMI and REPLACE-2 trials. The HORIZONS-AMI, ISAR-REACT-4, and REPLACE-2 trials all utilized bivalirudin with 0.7 mg/kg bolus followed by an infusion of 1.75 mg/kg/hour for the procedure duration. In ACUITY, patients received a bolus dose of bivalirudin was 0.5 mg/kg. In the BRIGHT trial, bivalirudin was continued at a dose of 0.25 mg/kg/hour no more than 4 hours afer PCI. Clopidogrel was used as the main P2Y12 inhibitor in all trails. All trials reported outcomes at 30 days. S1 Table provides measures of study quality.

**Table 1:** Characteristics of the included studies.

Study	Year	Type of Patients	Patients (n)	Anticoagulation		P2Y12 Inhibitors
				Heparin+GPI	Bivalirudin	
**BRIGHT[12]**	2016	NSTEMI/STEMI	392	60 IU/kg bolus	0.75 mg/kg bolus with 1.75 mg/kg/h	Clopidogrel
				tirofiban(10 μg/kg boluses with0.15 μg/kg/min)		
**HORIZONS-AMI[13]**	2015	STEMI	842	60 IU/kg bolus	0.75 mg/kg bolus with 1.75 mg/kg/h	Clopidogrel
				abciximab(0.25 mg/kg bolus with 0.125 μg/kg/min)	tirofiban/eptifibatide(provisional)	
				eptifibatide(180 μg/kg bolus plus 2.0 μg/kg/min)		
**ISAR-REACT4[14]**	2013	NSTEMI	399	70 IU/kg	0.75 mg/kg bolus with 1.75 mg/kg/h	Clopidogrel
				abciximab(0.25 mg/kg bolus with 0.125 μg/kg/min)		
**ACUITY[15]**	2009	NSTEMI	1401	60 IU/kg blous	0.5 mg/kg bolus with 1.75 mg/kg/h	Clopidogrel
				enoxaparin 1 mg/kg		
**REPLACE-2[16]**	2006	PCI	1537	65 IU/kg bolus	0.75 mg/kg bolus with 1.75 mg/kg/h	Clopidogrel
				enoxaparin 0.5 mg/kg; abciximab(0·25 mg/kg bolus with 0·125 μg/kg/min)	enoxaparin/abciximab(provisional)	

GPI = Glycoprotein Platelet IIb/IIIa inhibitor; NSTEMI = Non-ST-elevation myocardial infarction; STEMI = ST-elevation myocardial infarction; PCI = percutaneous coronary intervention.

### Efficacy Outcomes

**Fig 2:**
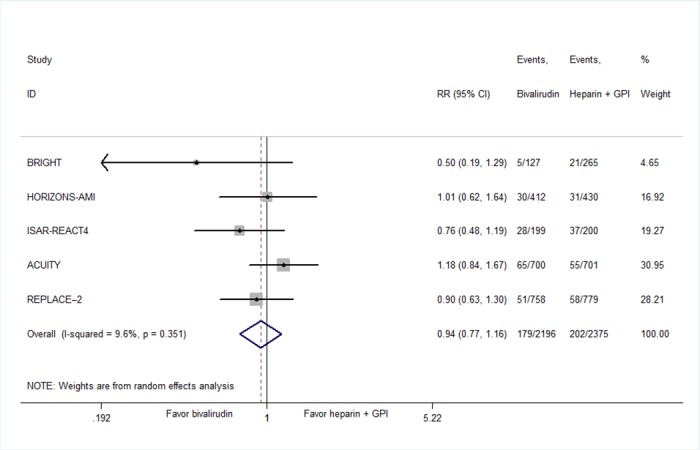
Summary plot of MACEs for bivalirudin versus heparin plus GPI. Squares or diamonds to the left of the solid vertical line indicate benefit with bivalirudin. CI = confidence interval; RR = risk ratio; MACE = major adverse cardiovascular event; GPI = glycoprotein IIb/IIIa inhibitor.

**Fig 3:**
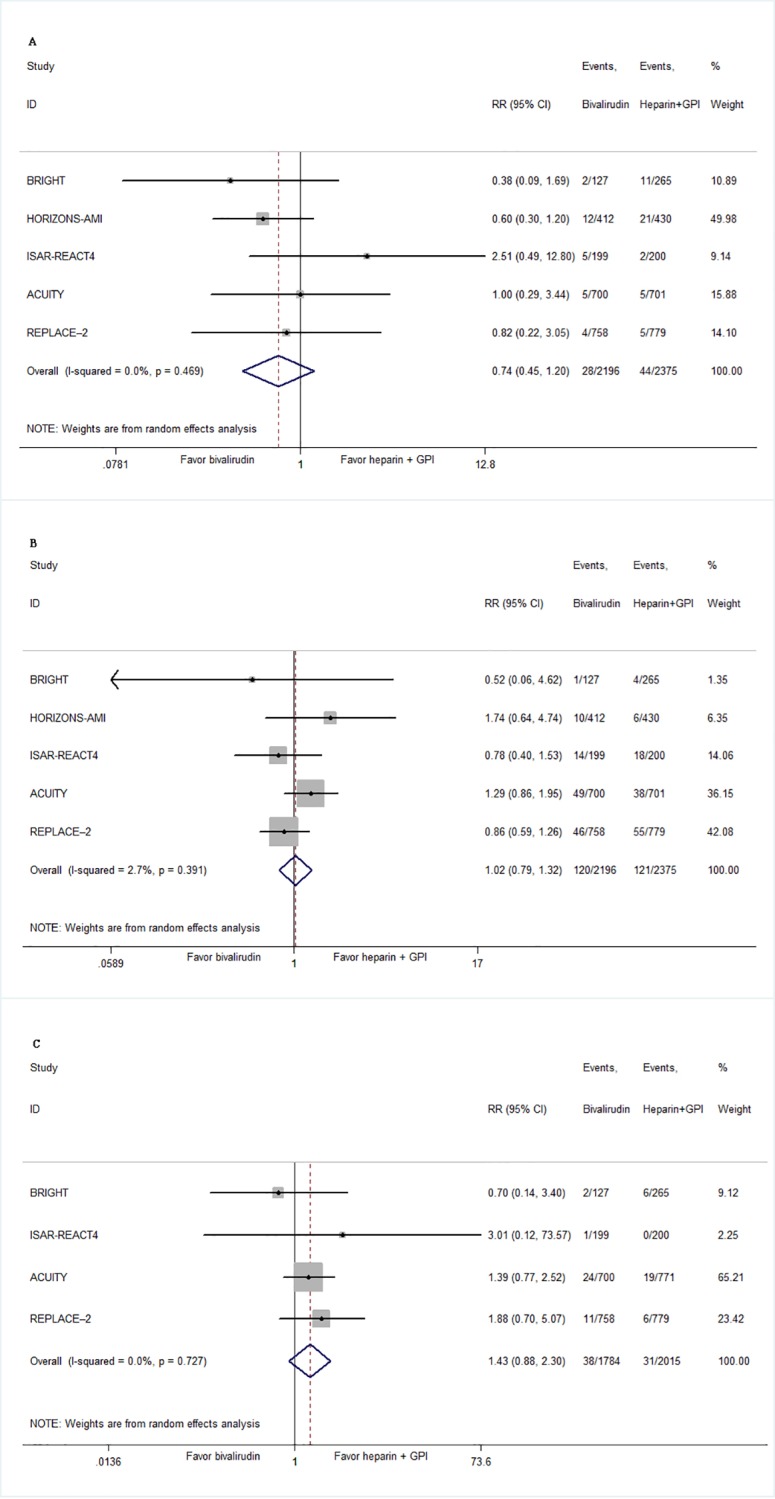
**Summary plot of bivalirudin and risk of all-cause mortality(A), myocardial infarction (B), urgent/ischemia-driven revascularization of target vessel(C).** Squares or diamonds to the left of the solid vertical line indicate benefit with bivalirudin. CI = confidence interval; RR = risk ratio; MACE = major adverse cardiovascular event.

No significant difference in MACE emerged between bivalirudin and heparin plus GPI at 30 days (8.15% vs 8.76%, RR 0.94, 95% CI 0.77–1.16, *P* = .57; Fig 2). There was no significant heterogeneity between the trials (I^2^ = 9.7%, *P* = .35). At 30 days, there were no significant differences in rates of mortality (1.28% vs 1.91%, RR 0.73, 95% CI 0.45–1.20, *P* = .22; Fig 3A), MI (5.46% vs 5.25%, RR 1.02, 95% CI 0.79–1.32, *P* = .88; Fig 3B), or target vessel revascularization (2.13% vs 1.65%, RR 1.43, 95% CI 0.88–2.30, *P* = .15; Fig 3C). No significant heterogeneity was seen for these results. These findings were consistent regardless of the type of patients the trials enrolled (Figure A in S1 File). There was no difference in MACE at 30 days between bivalirudin and heparin plus GPI (6.74% vs 7.46%, RR 0.90, 95% CI 0.63–1.30, *P* = .58) in REPLACE-2 trial that enrolled patients undergoing urgent or elective PCI. Similar result was obtained in other four trails enrolled patients with STEMI or NSTEMI (8.90% vs 9.02%, RR 0.93, 95% CI 0.70–1.25, *P* = .65). Summary results for MACE did not change by excluding each individual study in sensitivity analyses (Figure B in S1 File).

### Safety outcomes

**Fig 4:**
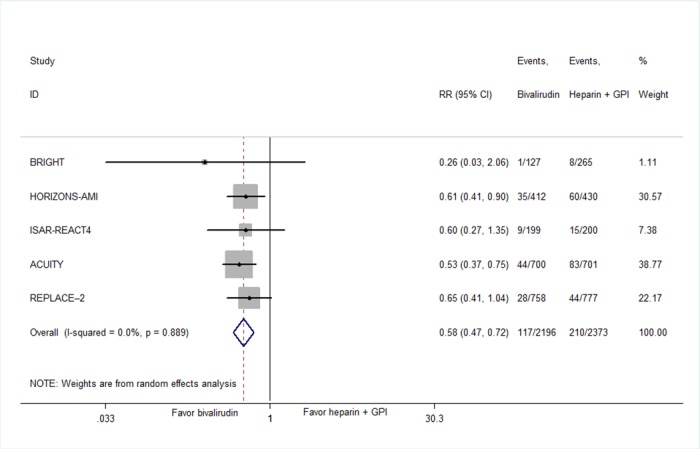
Summary plot of major bleeding for bivalirudin versus heparin plus GPI. Squares or diamonds to the left of the solid vertical line indicate benefit with bivalirudin. CI = confidence interval; RR = risk ratio; MACE = major adverse cardiovascular event.

Compared with heparin plus GPI, bivalirudin was associated with a significant reduction in 30-day major bleeding (5.32% vs 9.20%, RR 0.58, 95% CI 0.47–0.72, *P* < .0001; Fig 4). In the subgroup analyses of concomitant GPI use in bivalirudin arm, there were no significant differences in patients receiving the bivalirudin monotherapy (9.55% vs 9.69%, RR 0.87, 95% CI 0.56–1.35, *P* = .54).The same results were observed in bivalirudin plus provisional GPI group (6.92% vs 7.36%, RR 0.94, 95% CI 0.70–1.26, *P* = .68; Figure C in S1 File). The reduced hazard of major bleeding with bivalirudin versus heprin plus GPI was observed in majority femoral procedures (7.20% vs 11.90%, RR 0.61, 95% CI 0.43–0.87, *P* = .006), but not with majority racial procedures (0.78% vs 3.01%, RR 0.26, 95% CI 0.33–2.06, *P* = .20).

### Publication Bias

Visual inspection of funnel plots did not suggest any publication bias (Figure D in S1 File). There was no publication bias for MACE by Begg’s test and Egger’s test (*P* = .22; *P* = .19, respectively).

## Discussion

In this meta-analysis, of 4501 female patients enrolled in five RCTs, we compared bivalirudin with heparin plus GPI in female patients who underwent PCI. At 30 days, bivalirudin therapy in women was not associated with a reduction in the incidence of MACE in comparison with heparin plus GPI. The rates of all-cause mortality, MI, urgent/ischemia-driven revascularization of target vessel were similar in both groups. Bivalirudin substantially decreased the risk of major bleeding overall. When GPI use was planned in the heparin arm, the risk of major bleeding was 42% lower in the bivalirudin arm than in the heparin arm. In this meta-analysis, bivalirudin decreased the 30-day incidence of major bleeding in all trials, except ISAR-REACT-4 and BRIGHT. One possible explanation is that the high risk NSTEMI population (mean age = 71 years and rate of 3-vessel coronary artery disease = 50%) included in the ISAR-REACT-4 trial. In BRIGHT trail, there was no significant reduction in major bleeding. Only 49.4% female patinets received GPI in the heparin arm was one of the reasons. Another explanation was that the low rate of major bleeding for most patients undergoing PCI with radial access. Subgroup analyses demonstrated that no significant difference between the type of patients the trials enrolled in terms of reducing the risk of MACEs. To the best of our knowledge, this is the largest meta-analysis to evaluate the 30-day safety and efficacy of bivalirudin compared with those of heparin plus GPI in female patients undergoing PCI.

Female gender has been identified to be at higher risk than men for mortality and perioperative bleeding in numerous studies [5,17–19]. However, gender was not an independent predictor of mortality [20,21]. Previous reports have suggested that the worse outcomes in female patients undergoing PCI would be associated with more cardiovascular risk factors in women compared to men[18,22]. Another topic to be addressed is that sex-specific differences in pharmacologic response to antithrombotic drugs [23]. The optimal antithrombotic regimen of reduing the occurrence of bleeding without increasing ischemic events for female patients remains unknown. Bivalirudin has been regarded as a safe and effective anticoagulation therapy in AMI patients undergoing PCI [8,24,25]. On the contrary, the recently published HEAT-PPCI (How Effective are Antithrombotic Therapies in Primary PCI) trial [26] reported that bivalirudin compared with heparin alone resulted in a significantly higher rate of major adverse ischemic events with no decrease in bleeding complications. The diverging results among RCTs demonstrated that concomitant administration of a GPI with heparin might influence the benefit of bivalirudin [27]. Whether bivalirudin decreased the risk of bleeding regard less of routine GPI use remains unknow. The BRIGHT trail and EUROMAX trial showed that bivalirudin decreased the risk of bleeding regardless of GPI use when compared to heparin. The previous meta analysis [28] clarified that the benefit of bivalirudin in reducing major bleeding compared with heparin depends on the routine rather than provisional use of GPI. Whether the addition of GPI imparts and accentuated bleeding risk for women (rather than men) is unclear. As demonstrated in this analysis, bivalirudin contributed to a 42% reduction in major bleeding compared to heparin plus GPI. Bivalirudin therapy in women is safe, resulting in a lower rate of major bleeding when compared to heparin plus GPI no matter whether GPI use was provisional in the bivalirudin arm in this analysis.

The largest trial to date comparing radial with femoral access for patients with ACS undergoing PCI showed radial access reduced the major bleeding [29]. Several meta- analyses also support the benefits of radial access PCI in ACS [30,31]. In this analysis, the reduced hazard of major bleeding with bivalirudin versus heprin plus GPI was observed in majority femoral access, but not with racial. However, the results of MATRIX showed that the major bleeding reduction in the bivalirudin group is not attributable to radial access [25]. The rate of MACE was not significantly lower with bivalirudin arm than with heparin arm in MATRIX study, which may have association with reduced doses of bivalirudin as described in detail previously[32]. Whether radial access might add any benefit compared with femoral in patients treated with bivalirudin remain unanswered. The ongoing SAFARI-STEMI (the Safety and Efficacy of Femoral Access Versus Radial for Primary Percutaneous Coronary Intervention in ST-Elevation Myocardial Infarction) trial (NCT01398254) will help shed light into this particular topic.

Multiple evidence demonstrates that increased safety of peri-PCI antithrombotic therapy can have a profound impact on short- and long-term mortality [32,33]. When comparing bivalirudin and heparin plus GPI, we found no difference in 30-day mortality overall. The similar result was obsreved in a patient-level pooled analysis in women [34]. Given only two studies reported the 1-year mortality, we did not perform a pooled analysis of 1-year mortality between bivalirudin arm and heparin plus GPI arm. However, the 1-year mortality was no different between two arms both in the ACUITY and REPLACE-2 trials. There were no significant differences in the incidences of MACE, MI and target vessel revascularization in female patients undergoing PCI treated with bivalirudin compared with heparin plus GPI. The 3-year follow up data from HORIZON-AMI demostrated that the absolute reduction in mortality with bivalirudin monotherapy not only persisted with long-term follow-up, but also continued to increase with time, which was consistent with delayed benefits from prevention of major bleeding[35].

Some reports have suggested that bivalirudin substantially increased the risk of acute stent thrombosis (AST) in patients with STEMI [36,37]. In our analysis, only the BRIGHT trail reported the stent thrombosis rate and no difference existed between two treatment arms. The authors explained that low rates of acute stent thrombosis in BRIGHT might be associated with patients in bivalirudin arm received a post-procedure PCI bivalirudin infusion. The same results were reported from the EUROMAX trial. A Bayesian network meta-analysis[38] also showed that bivalirudin increased the risk of AST after primary PCI, which may be mitigated by continuing a full PCI dose of bivalirudin 3 to 4 h post-PCI. For STEMI patients, complete revascularizarion is potentially a beneficial strategy. There is a wealth of observational data support complete revascularizarion, while the RCTs suggest the opposite. Recently, a meta- analysis of Moretti et al. demonstrated that complete revascularization strategy in STEMI patient appears safe at short term and offers a reduction in repeated revascularization at1 year[39]. Although most of the data of this meta-analysis derived from the non RCTs, complete revascularization performed during primary PCI for patients presenting with STEMI is inferior to culprit-only approach. Patients with diabetes mellitus (DM) undergoing PCI result in worse long-term survival compared with patients without DM. Much less is known that bivalirudin is safe and effective in DM patients. At present, the studies that compared bivalirudin with heparin plus GPI in female patients with DM undergoing PCI are lacking. The results of a meta-analysis[40] showed that thrombin inhibition with bivalirudin alone was associated with reduced 30-day major bleeding and 1-year all-cause mortality compared with heparin plus GPI in DM patients undergoing PCI. Further investigations will be needed to verify the benefits of bivalirudin in female patients with DM undergoing PCI.

Contrary to other meta-analyses [41] on a wider scale of patients undergoing PCI that tended to show trends of higher ischemic events while markedly lower bleeding complications, the results of this analysis suggest both efficacy and safety are maintained with bivalirudin monotherapy in women udergoing PCI. The findings of this meta-analysis concured with a patient-level meta-analysis[34] showing a more pronounced clinical benefit of bivalirudin in women undergoing PCI including a significant reduction in major bleeding and a significant reduction in mortality rates at 1 year. Further studies are needed to definitively confirm the benefit of bivalirudin in women.

Limitations of our meta-analysis were the results of this meta-analysis were derived from study-level data of published subgroup analyses of randomized trials and were not based on individual patient data. Also, although potential confounders were adjusted for, the nature of nonrandomized subgroup analysis may generate publication bias. Moreover, the different protocols and characteristics may cause heterogeneity. However, the sensitivity analyses showed that no single study affected the result of MACE, suggesting that the overall effect is robust and justified. Overall, we were limited to present only 30-day outcomes for most of the included trials did not report 1-year follow-up data.

## Conclusion

In female patients undergoing PCI, a bivalirudin based regimen compared with a heparin plus GPI based regimen is associated with a significant reduction in 30-day major bleeding without increased ischemic events.

## Supporting Information

S1 PRISMA Checklist Preferred Reporting Items for Meta-Analyses (PRISMA) statement checklist. (DOC)

S1 File Supporting Information Figures. Figure A. Subgroup analysis: Summary plot of MACEs for the type of patients the trials enrolled. Squares or diamonds to the left of the solid vertical line indicate benefit with bivalirudin. CI = confidence interval; RR = risk ratio; MACE = major adverse cardiovascular event; GPI = glycoprotein IIb/IIIa inhibitor. Figure B. Sensitivity analyses for MACEs. CI = confidence interval. Figure C. Subgroup analysis: Summary plot of MACEs for concomitant GPI use in bivalirudin arm. Squares or diamonds to the left of the solid vertical line indicate benefit with bivalirudin. CI = confidence interval; RR = risk ratio; MACE = major adverse cardiovascular event; GPI = glycoprotein IIb/IIIa inhibitor. Figure D. Funnel plot of included studies. The oblique line in the center is the natural logarithm of pooled relative risk, and the 2 solid lines are pseudo 95% confidence limits.
(ZIP)

S1 Table Quality assessment of included study. (DOC)
