# Supplementary figures and images for: Bivalirudin versus Heparin plus Glycoprotein IIb/IIIa Inhibitors in Women Undergoing Percutaneous Coronary Intervention: A Meta-Analysis of Randomized Controlled Trials
Source: PLoS One. 2017 Jan 17;12(1):e0169951. doi: 10.1371/journal.pone.0169951 (PMC5241007; doi:10.1371/journal.pone.0169951)

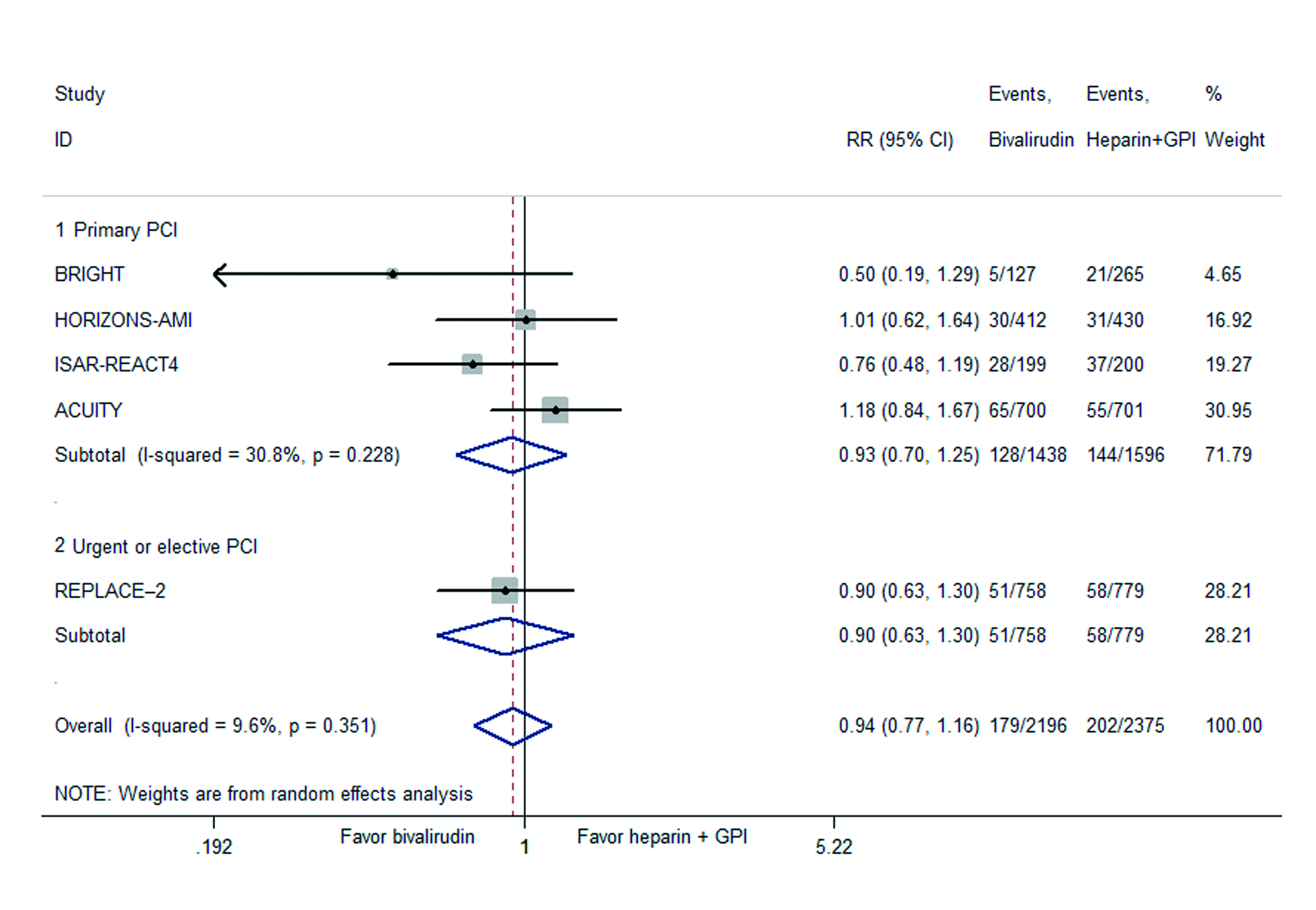

Supplement: S1 File — Figure A. Subgroup analysis: Summary plot of MACEs for the type of patients the trials enrolled. Squares or diamonds to the left of the solid vertical line indicate benefit with bivalirudin. CI = confidence interval; RR = risk ratio; MACE = major adverse cardiovascular event; GPI = glycoprotein IIb/IIIa inhibitor. Figure B. Sensitivity analyses for MACEs. CI = confidence interval. Figure C. Subgroup analysis: Summary plot of MACEs for concomitant GPI use in bivalirudin arm. Squares or diamonds to the left of the solid vertical line indicate benefit with bivalirudin. CI = confidence interval; RR = risk ratio; MACE = major adverse cardiovascular event; GPI = glycoprotein IIb/IIIa inhibitor. Figure D. Funnel plot of included studies. The oblique line in the center is the natural logarithm of pooled relative risk, and the 2 solid lines are pseudo 95% confidence limits. (ZIP) [file pone.0169951.s002.zip › S1 file/Figure A.tif]

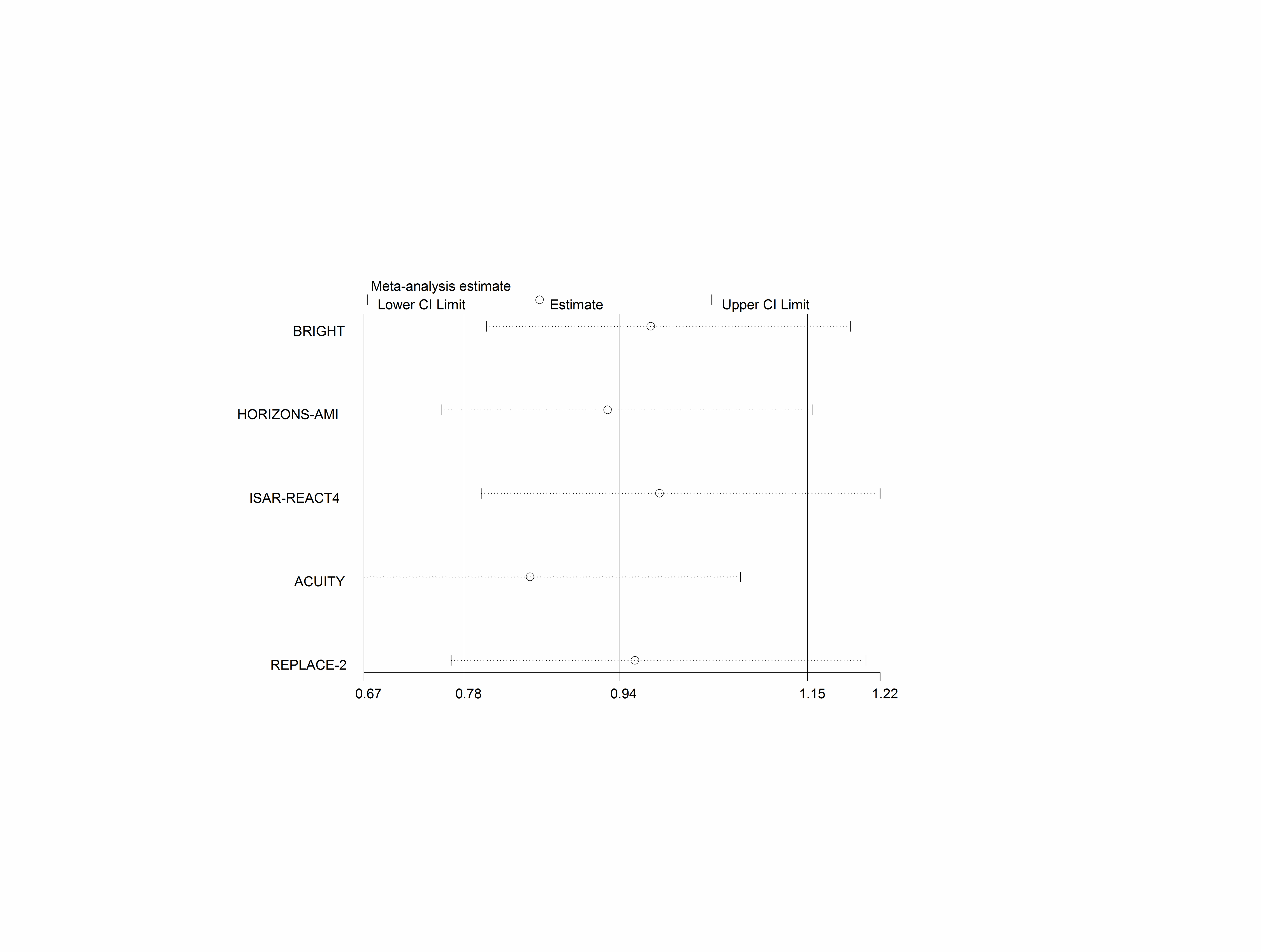

Supplement: S1 File — Figure A. Subgroup analysis: Summary plot of MACEs for the type of patients the trials enrolled. Squares or diamonds to the left of the solid vertical line indicate benefit with bivalirudin. CI = confidence interval; RR = risk ratio; MACE = major adverse cardiovascular event; GPI = glycoprotein IIb/IIIa inhibitor. Figure B. Sensitivity analyses for MACEs. CI = confidence interval. Figure C. Subgroup analysis: Summary plot of MACEs for concomitant GPI use in bivalirudin arm. Squares or diamonds to the left of the solid vertical line indicate benefit with bivalirudin. CI = confidence interval; RR = risk ratio; MACE = major adverse cardiovascular event; GPI = glycoprotein IIb/IIIa inhibitor. Figure D. Funnel plot of included studies. The oblique line in the center is the natural logarithm of pooled relative risk, and the 2 solid lines are pseudo 95% confidence limits. (ZIP) [file pone.0169951.s002.zip › S1 file/Figure B.tif]

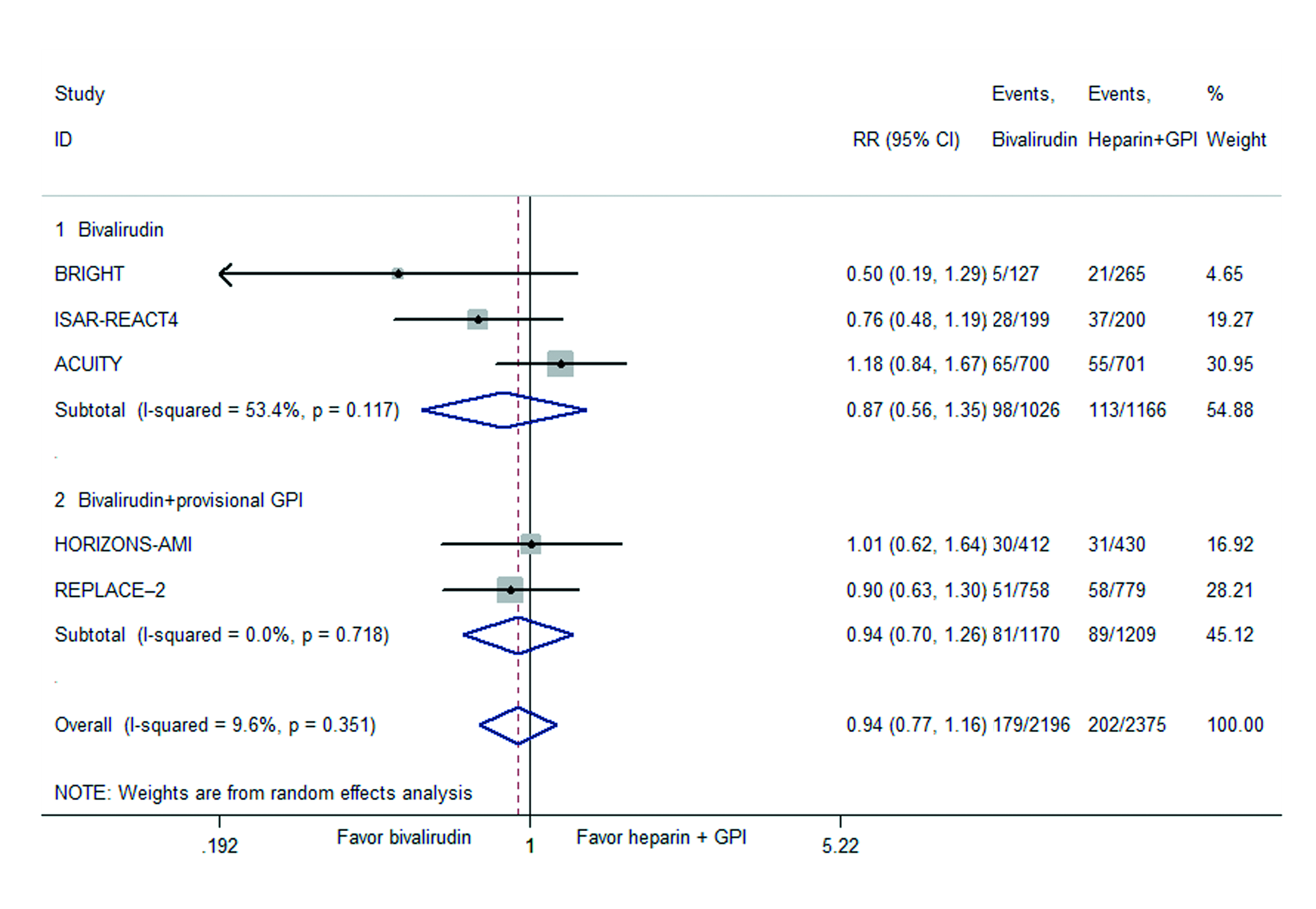

Supplement: S1 File — Figure A. Subgroup analysis: Summary plot of MACEs for the type of patients the trials enrolled. Squares or diamonds to the left of the solid vertical line indicate benefit with bivalirudin. CI = confidence interval; RR = risk ratio; MACE = major adverse cardiovascular event; GPI = glycoprotein IIb/IIIa inhibitor. Figure B. Sensitivity analyses for MACEs. CI = confidence interval. Figure C. Subgroup analysis: Summary plot of MACEs for concomitant GPI use in bivalirudin arm. Squares or diamonds to the left of the solid vertical line indicate benefit with bivalirudin. CI = confidence interval; RR = risk ratio; MACE = major adverse cardiovascular event; GPI = glycoprotein IIb/IIIa inhibitor. Figure D. Funnel plot of included studies. The oblique line in the center is the natural logarithm of pooled relative risk, and the 2 solid lines are pseudo 95% confidence limits. (ZIP) [file pone.0169951.s002.zip › S1 file/Figure C.tif]

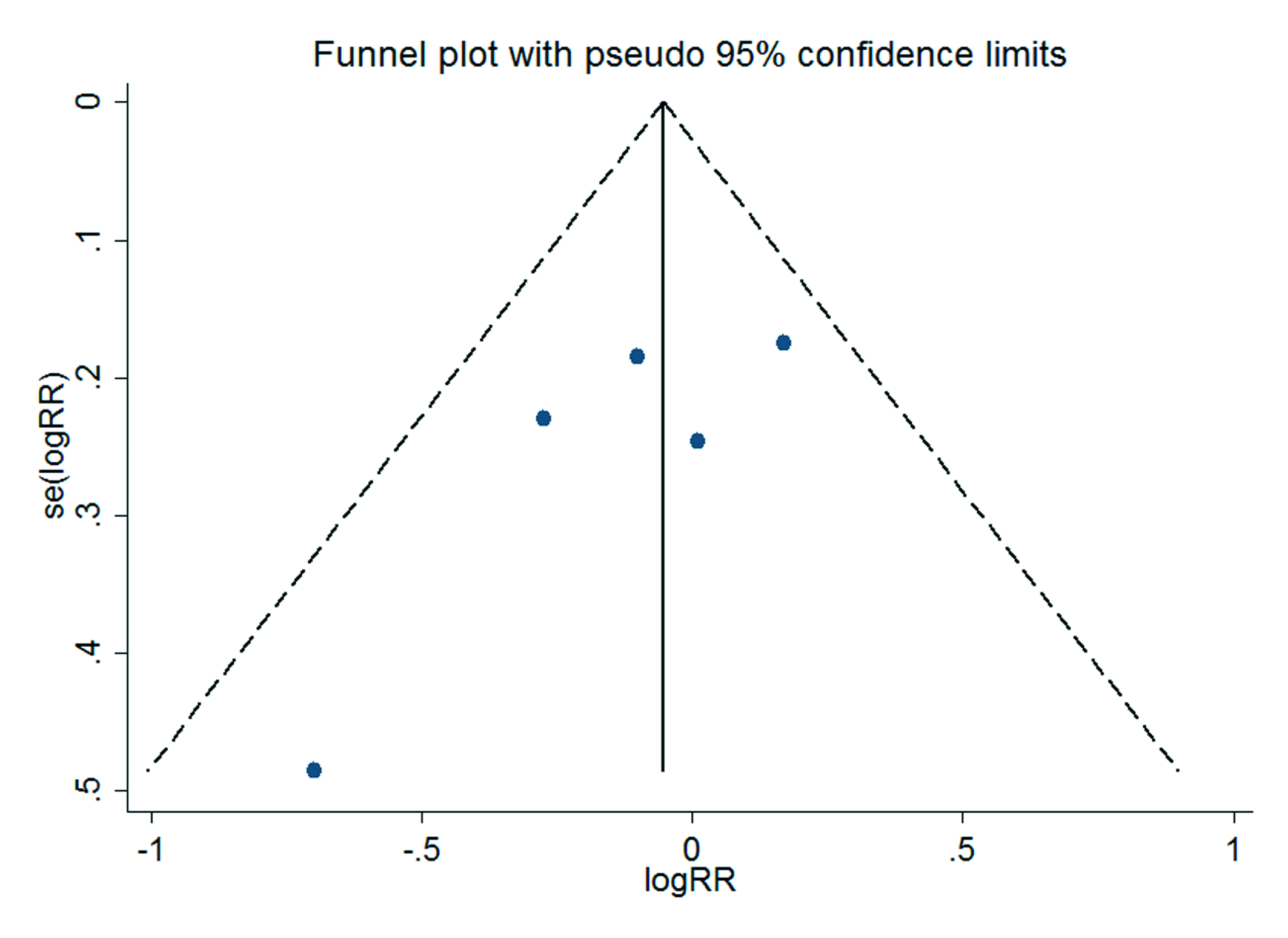

Supplement: S1 File — Figure A. Subgroup analysis: Summary plot of MACEs for the type of patients the trials enrolled. Squares or diamonds to the left of the solid vertical line indicate benefit with bivalirudin. CI = confidence interval; RR = risk ratio; MACE = major adverse cardiovascular event; GPI = glycoprotein IIb/IIIa inhibitor. Figure B. Sensitivity analyses for MACEs. CI = confidence interval. Figure C. Subgroup analysis: Summary plot of MACEs for concomitant GPI use in bivalirudin arm. Squares or diamonds to the left of the solid vertical line indicate benefit with bivalirudin. CI = confidence interval; RR = risk ratio; MACE = major adverse cardiovascular event; GPI = glycoprotein IIb/IIIa inhibitor. Figure D. Funnel plot of included studies. The oblique line in the center is the natural logarithm of pooled relative risk, and the 2 solid lines are pseudo 95% confidence limits. (ZIP) [file pone.0169951.s002.zip › S1 file/Figure D.tif]
