# Supplementary material for: Bivalirudin versus Heparin plus Glycoprotein IIb/IIIa Inhibitors in Women Undergoing Percutaneous Coronary Intervention: A Meta-Analysis of Randomized Controlled Trials
Source: PLoS One. 2017 Jan 17;12(1):e0169951. doi: 10.1371/journal.pone.0169951 (PMC5241007; doi:10.1371/journal.pone.0169951)
Supplement: S1 Table — (DOC) [file pone.0169951.s003.doc]

**S1 Table. Quality assessment of included study**

| Study(ref#) | Sequence generation | Allocation concealment | Blinding | | Incomplete outcome data | Selective reporting | Other bias | Single/ Multicenter | Jadad  scores |
| --- | --- | --- | --- | --- | --- | --- | --- | --- | --- |
|  |  |  | participants and personnel | outcome assessment |  |  |  |  |  |
| BRIGHT[12] | Sealed envelopes with | Sealed envelopes with | Open label | Blinding | Adequate | No | Unclear | Multicenter | 5 |
|  | a block size of 6 | a block size of 6 |  |  |  |  |  |  |  |
| HORIZONS-AMI[13] | Dynamic(minimization) | Telephone randomization by | Open label | Blinding | Adequate | No | Unclear | Multicenter | 5 |
|  | allocation scheme | a computerized, interactive |  |  |  |  |  |  |  |
|  |  | voice-response system |  |  |  |  |  |  |  |
| ISAR-REACT4[14] | Randomized | Sealed opaque | Double | Blinding | Adequate | No | Unclear | Multicenter | 6 |
|  | assignment | envelopes |  |  |  |  |  |  |  |
| ACUITY[15] | Generator in blocks of | Assigned with an interactive | Open label | Blinding | Adequate | No | Unclear | Multicenter | 5 |
|  | six stratified by site | voice response system |  |  |  |  |  |  |  |
| REPLACE-2[16] | Randomized | Central telephone | Double | Blinding | Adequate | No | Unclear | Multicenter | 6 |
|  | assignment | system |  |  |  |  |  |  |  |
